# Sterilization regret in India: Is quality of care a matter of concern?

**DOI:** 10.1186/s40834-020-00115-8

**Published:** 2020-09-08

**Authors:** Anjali Bansal, Laxmi Kant Dwivedi

**Affiliations:** 1 International Institute for Population Sciences, Mumbai, 400088 India; 2 Department of Mathematical Demography and Statistics, International Institute for Population Sciences, Mumbai, 400088 India

**Keywords:** Sterilization, Regret, Quality of care, Health facility, Public, Private

## Abstract

**Background:**

According to United Nations, 19% of females in the world relied only on the permanent method of family planning, with 37% in India according to NFHS-4. Limited studies tried to measure the sterilization regret, and its correlated factors. The study tried to explore the trend of sterilization regret in India from 1992 to 2015 and to elicit the determining effects of various factors on sterilization regret, especially in context to perceived quality of care in the sterilization operations and type of providers.

**Data and methods:**

The pooled data from NFHS-1, NFHS-3 and NFHS-4 was used to explore the regret by creating interaction between time and all the predictors. Predicted probabilities were calculated to show the trend of sterilization regret amounting to quality of care, type of health provider at the three time periods.

**Results:**

The sterilization regret was increased from 5 % in NFHS-1 to 7 % in NFHS-4. According to NFHS-4, for those whose sterilization was performed in private health facility the regret was found to be less (OR-0.937; 95% CI- (0.882–0.996)) compared to public health facility. Also, the results show a two-fold increase in regret when women reported bad quality of care. The results from predicted probabilities provide enough evidence that the regret due to bad quality of care in sterilization operation had increased with each subsequent round of NFHS.

**Conclusion:**

Many socio-economic and demographic factors have influenced the regret, but the poor quality of care contributed maximum to the regret from 1992 to 2015. The health facilities have seriously strayed from improving the health and well-being of women in providing the family planning methods. In addition, to public facilities, the regret amounting to private facilities have also increased from NFHS-1 to 4. The quality of care provided in the family planning operation should be standardized in every hospital to strengthen the health systems in the country. The couple should be motivated to adopt more of spacing methods.

## Introduction

India was the first country to launch its family planning programme in 1952 to control the population [26]. During the programme, the government made available many contraceptive methods to the couples like condoms, IUD, diaphragm, and sterilization [37]. The method of sterilization gained popularity soon after the implementation and during the emergency period (1975–77) around 8 million sterilizations were reported [38], where majority of them were forced and performed on men. Due to the mass “forced” sterilization, the family planning programme approach shifted to family welfare approach, and male sterilization almost disappeared from the family planning programme [29, 43] and female sterilization emerged as the only permanent method of contraception in the country.

According to the UN, 19% of married or in-union women in the world relied on female sterilization [52]. In India, during 2014–2015, more than 4 million sterilizations were done [34]; out of which only 1 lakh were performed on men [4]. The latest estimates provided by NFHS –4 (2015–2016), also showed the similar picture where 37% of currently married women in India relied on the female sterilization [16].

Sterilization is a permanent method which cannot be reversed, so it should be performed only after been informed about the side effects and consequences of the same [39, 44]. About 10% women worldwide experienced regret because of the sterilization [10, 14, 44, 54], and in India according to NFHS-3 (2005–2006), around 5 % women regretted their decision of sterilization [17]. Different research on sterilization regret stated that many women regretted about the routine process due to the various socio-economic variables [6, 15, 30], child loss post sterilization [13, 21, 28, 44], quality of care and type of health provider [23, 44]. In 1988, Donabedian [8] defined quality of health care based on the performance of practitioners, care provided in the health systems and whether effective care is sought. Later based on these lines, Bruce [5], devised a framework for family planning services that majorly focused on the needs of the couples rather than demographic outcomes. Later this framework was referred as Bruce/Jain Framework, they have suggested six elements to address the quality of care issues, choice of methods, information given to clients, intra-personal relationship of clients and providers, technical competence of providers, follow-up or continuity mechanism, and appropriate constellations of services [5, 18]. In India, different studies addressing the issue of quality provided in the family planning operations were studied well extensively, but all studies in a way concluded that the quality in the family planning operations lacks the major dimensions of quality of care suggested by Bruce/Jain. In India, a review done by Koeing, Foo and Joshi [23], suggested that geographical variability exists in service delivery (lower levels of provider-client contact, infrastructure support and rapport and affinity between clients and service providers) especially in the Northern India.

There had been extensive literature in and around the world addressing the issue of the female sterilization and but only few studies tried to measure regret out of it. The study tried to explore the trend and pattern of sterilization regret in India from 1992 to 2015 and to elicit the determining effects of various factors on post sterilization regret in women, especially in context to perceived quality of care in the sterilization operations and type of providers. In NFHS, there was no question asked on the provider competence, client/provider relations, re-contact and follow up mechanism and on appropriate constellation of services. So we have used the perceived quality of care reported by women as per their experience of sterilization operation.

## Data and methods

### Data

The present study used data from the three rounds of National Family Health Survey (NFHS),^1^ first was conducted in 1992–1993, third was conducted in 2005–2006 and the fourth in 2015–2016. NFHS is a nationally representative cross-sectional survey which includes representative samples of households throughout India. The survey provides state, and national level estimates of demographic and health parameters as well as data on various socio-economic and program dimensions, which are critical for implementing the desired changes in demographic and health parameters. A two-stage stratified sample was collected in NFHS-4 from 29 states and seven union territories (for detailed sampling see [16]). The survey for the first time in 2015–16 provided district-level estimates on the various key indicators associated with the demographic and health parameter for the country. The NFHS-1 interviewed 88,562 households and 89,777 ever-married women in the age group 13–49 from 24 states and Delhi. The NFHS-3 interviewed 109,041 households, 124,385 women age 15–49, and 74,369 men aged 15–54. In comparison, NFHS-4 interviewed 601,509 households, 699,686 women age 15–49, and 112,122 men aged 15–54. Since the objective of the paper was to examine the post sterilization regret, we have filtered only those women who have reported being sterilized at the time of the survey. In NFHS-1 23,136 women, in NFHS-3 32,519 women and in NFHS-4 165,276 women were reported to be sterilized.

### Methods

**Fig. 1:**
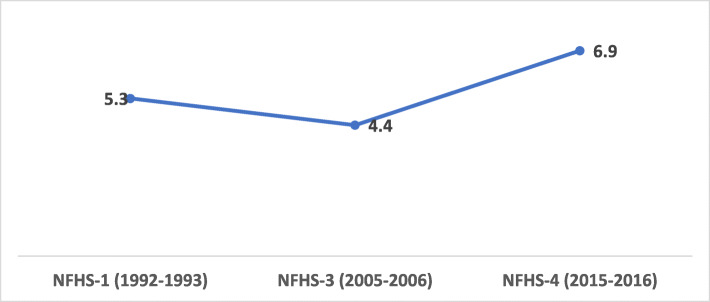
Sterilization regret trend from NFHS-1(1992–1993) to NFHS-4(2015–2016), India

In this study, we have pooled the three rounds of NFHS; NFHS-I (1992–1993), NFHS-III (2005–2006) and NFHS-IV (2015–2016). All the dummy variables were interacted with the time period of the survey. The estimates of the different rounds of NFHS were comparable because of its sampling design [31, 42]. Many studies in the past have pooled different DHS/NFHS rounds to observe the trends over time [20, 31, 40, 42] (Fig. 1).

**Fig. 2:**
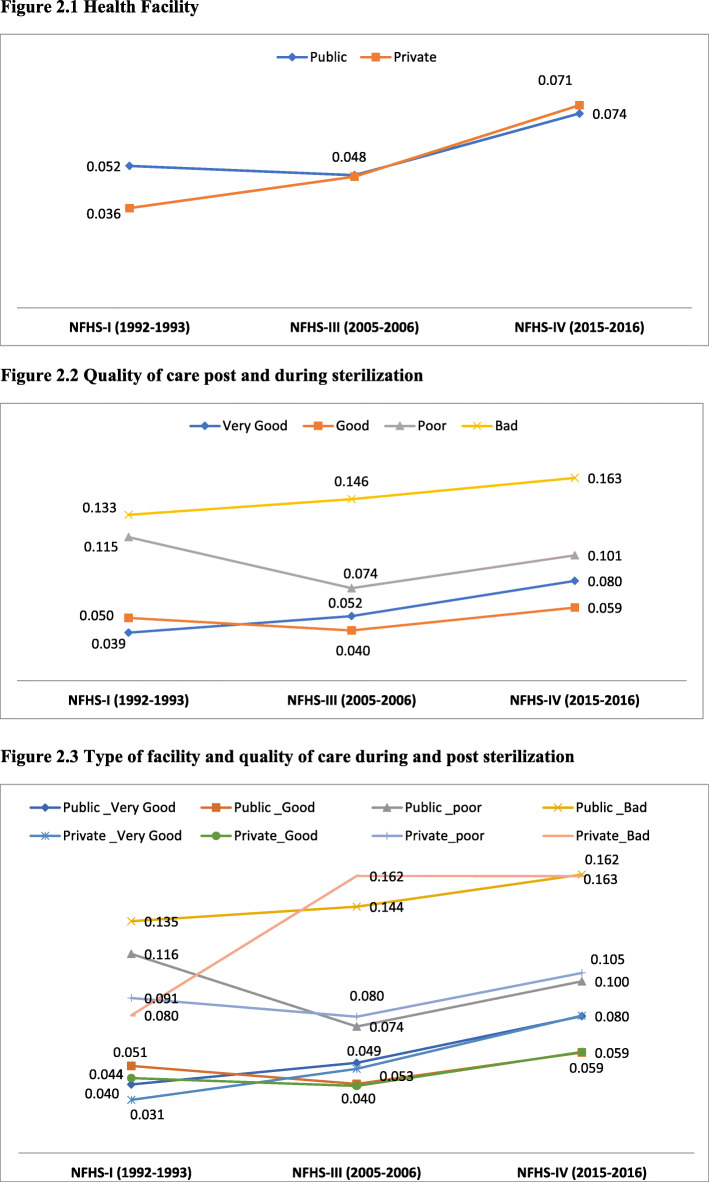
Predicted Probabilities for women who reported sterilization regret, by the Quality of care post sterilization, Type of health Facility, NFHS-I (1992–1993), NFHS-III (2005–2006) and NFHS-IV (2015–2016). **Figure 2.1** Health Facility. **Figure 2.2** Quality of care post and during sterilization. **Figure 2.3** Type of facility and quality of care during and post sterilization

To measure the sterilization regret among the sterilized ever-married women over the time period, we have fitted a pooled binary logistic regression analysis. Descriptive and univariate analyses using logistic regression were performed on the latest round of NFHS (2015–16) to explore the regret among the women. The effects of quality of care and type of provider providing the care on the sterilization regret were explored. Initially based on growing literatures on sterilization, we have selected 18 covariates, and univariate logistic analysis were performed to select independent variables for the multivariate model. Factors found in univariate analysis (Table 1) to be significantly associated (*P*-value < 0.05) were included in the multivariate model. In the pooled binary logistic regression model, the interaction between time of the survey and all the predictors variables were created, and the results of this analysis have been presented as a set of predicted probabilities of being regretting about sterilization by two categories of type of health provider, and four categories of quality of care during and post sterilization operation (Fig. 2). The advantage of using binary logistic regression procedure is that it models the log of the odds of an outcome occurring in terms of a vector of independent variables. The model in the study is defined as:

**Table 1:** Trends of sterilization regret among ever-married women in India and number of sterilized women by background variables, NFHS, 1992–2016

	NFHS-I(1992–1993)		NFHS-III(2005–2006)		NFHS-IV (2015–2016)	
Background variables	Percent regret	Total No. of sterilized women	Percent regret	Total No. of sterilized women	Percent regret	Total No. of sterilized women
**Regions**						
Classified by TFR more than 2.1	7.8	2517	4.5	15,831	6.4	73,320
Classified by TFR less than or equal to 2.1	5.1	19,910	4.3	16,688	7.1	91,956
**Place Of residence**						
Urban	4.0	7286	4.6	14,260	6.9	45,152
Rural	5.8	15,141	4.3	18,259	6.9	1,20,124
**Caste**						
Scheduled Caste/Tribe	5.8	4713	4.3	9344	6.8	57,211
Others	5.2	17,714	4.3	22,507	6.9	1,04,126
**Religion**						
Hindu	5.3	20,172	4.2	26,608	6.8	1,41,044
Muslim	8.0	1212	6.4	2784	8.6	11,230
Others	2.5	1043	4.1	3127	6.0	13,002
**Educational**						
No Education	5.7	12,084	4.3	14,406	6.6	71,249
Primary	6.0	5035	4.0	6063	6.8	28,584
Secondary	3.4	4808	4.8	10,689	7.3	58,584
Higher	4.0	500	4.8	1361	7.0	6859
**Wealth index**						
Poorest	8.0	2555	4.3	3417	6.6	29,245
Poorer	7.7	3025	4.2	4964	7.1	35,259
Middle	5.5	4572	4.3	6822	7.0	37,205
Richer	4.3	6122	4.5	8538	7.1	34,570
Richest	2.9	6153	4.6	8778	6.6	28,997
**Sex composition**						
No Male	14.2	961	8.0	2144	10.7	9876
1 Male	15.8	463	7.4	1292	9.7	8171
2+ Male	6.1	3229	4.8	5248	7.8	30,047
Both Male And Female	4.2	17,752	3.8	23,806	6.0	1,16,910
**Age at sterilization**						
< 29	5.5	15,806	4.6	25,562	7.1	1,23,830
30–39	4.4	6281	3.7	6746	6.4	38,890
40+	6.9	340	4.7	211	7.9	2556
**Parity at sterilization**						
Less Than 2	17.6	196	8.8	430	11.4	4720
More than 2	5.2	22,910	4.3	36,281	6.7	178,088
**Year since sterilization**						
Less Than 2	5.3	6503	3.6	6961	6.7	31,075
2–3	5.9	6929	4.8	7098	7.2	36,319
More than 3	4.8	8995	4.5	18,460	6.9	97,882
**Child loss post sterilization**						
No Loss	0.0	22,601	4.4	32,480	6.9	1,65,039
Male loss	0.0	4	10.8	15	12.6	154
Female Loss	0.0	2	3.3	24	20.1	83
**Quality of care**						
Very Good	4.1	11,656	4.7	16,908	7.8	78,891
All Right	5.6	9158	3.6	14,229	5.6	79,403
Not so good	11.7	1312	7.0	1226	9.5	6201
Bad	13.5	481	13.0	156	20.2	781
**Type Of health facility**						
Public	5.4	19,717	4.3	27,097	6.9	1,42,507
Private	4.2	2710	4.8	5422	6.8	22,769
**Total**	**5.3**	22,607	**4.4**	**32,519**	**6.9**	**1,65,276**

Percentages are weighted, N is non-weighted

$$ \log\ \left(\mathrm{Y}\right)=\mathrm{a}+{\mathrm{b}}_1{\mathrm{X}}_1+{\mathrm{b}}_2{\mathrm{X}}_2+{\mathrm{b}}_3{\mathrm{X}}_1{\mathrm{X}}_2 $$

Where log (Y) is the natural logarithm of the odds of the outcome (sterilization regret, binary variable), ‘a’ is the intercept and b_1_; b_2_ are the coefficients associated with each predictive variable, b_3_ is the coefficient associated with the interaction term of X_1_ and X_2._

The predicted probabilities were based on terms in the logistic regression model relating to interactions between year, type of health provider and quality of care. In the logistic regression model all the predictor variables are interacted with the year, but the predicted probabilities was calculated only for the variable of interest. All analyses were completed using Stata version 13, and all the results were reported at 5% level of significance.

### Variables

The dependent variable in the analysis was post sterilization regret, and it was coded as “0” if women do not report regret and “1” if reported regret. The independent variables were geographic regions^2^ (classified by TFR more than equal to 2.1 and classified by TFR less than 2.1), Place of residence (urban and rural), Caste of women (Scheduled Caste/Tribe and Others), Religion of the women (Hindu, Muslim and Others), Educational status of women (No Education, Primary, Secondary and Higher), Wealth Index (Poorest, Poorer, Middle, Richer, and Richest), Sex composition of living children (No Male,1 Male, 2+ Male, and both Male and Female), Age at sterilization(< 29, 30–39, and 40+), Parity at sterilization (Less than 2, 2–3, and more than 3), Year since sterilization(Less than 2, 2–3, and more than 3), Post sterilization child loss (No, Male loss, Female loss), Quality of care (very good, alright, not so good, bad) and Type of service provider (Public, Private, and others), compensation received for sterilization (Received and Not Received).

## Results

### Trend and differentials in sterilization regret by different predictors

The female sterilization users increased to 13% points from NFHS-1 to NFHS-4. The result indicated that the sterilization regret had increased from 6 % in NFHS I (1992–93) to 7 % in NFHS- IV (2015–16), so as the number of sterilized women (Fig. 1).

The maximum numbers of sterilized women were concentrated in the southern region of India, where maximum tubectomy was observed. In the southern region, a majority of Andhra Pradesh women adopted sterilization as the only method of family planning, but the highest regret percentage was found among North-eastern women, where maximum percentage was seen in Manipur in rounds of both NFHS-III and NFHS-IV. The least regret was seen in Himachal Pradesh in both rounds of NFHS. Table 1 represents the trends of sterilization regret among ever married women in India from 1992 to 2016. There had been an increase of 58% in overall sterilization regret from 2005 to 06 to 2015–16 (Appendix Tables 1 and 2). Table 1 shows that the percentage of users of sterilization had been increased to five times in a public health facility. The regret in the public facility had risen to 61% in the last decade. In NFHS-1 the regret in public facility was 5.4%, which decreases to 4.3% in NFHS-III, but again the regret amounting to sterilization conducted in public health facility was found to be 6.9%. The regret was also seen where the child was lost post-sterilization operation, though for NFHS-1 no women reported regret post child loss after sterilization. The quality of care proved to be one of the important determinant in explaining the regret among women, where maximum regret was found when the women reported bad quality of care in all the rounds of NFHS, and it had increased to 7 % points from NFHS-3 to NFHS-4. Place of the region also demonstrated a significant increase in the sterilization regret, where the regret was more in rural area than urban area in all three rounds of NFHS. Educational status and wealth Index also illustrated the same pattern, as a more impoverished and uneducated women experienced more regret than educated and wealthy women.

### Multivariate analysis

In order to examine the change in the magnitude of sterilization regret belonging to four categories of quality of care and two categories of service provider from 1992 to 2016, having adjusted the results for important socioeconomic and demographic characteristics, we ran a binary logistic regression model after pooling data from three rounds of NFHS. The addition of two-way interaction between the alright, not so good, and bad quality of care with the three variables of time were found to be statistically significant at 95% CI, also the two interaction between private health provider with the time period was also found to be statistically significant suggesting that the sterilization regret amounting the latter two variables have changed over time.

The predicted probabilities, presented in Fig. 2, suggests that the likelihood of sterilization regret among women attributed to bad quality of care during and post-sterilization increased from 1992 to 93 to 2015–16. Also, the probability of regret had increased more in the public health facility from NFHS-I to NFHS-IV, and majorly sterilizations conducted in public facilities (7.4%) were more regretting than done in the private facilities (7.1%). From the analysis it was evident that regret amounting to both private and public facilities had increased over the time. The figure provides enough evidence to suggest that the bad quality of care in sterilization operation had increased with each subsequent NFHS. The regret due to bad quality of care had increased from 13% in NFHS-1 to 16% in NFHS-4. This attributed that the care provided in the health facility deteriorated in a 23-year period gap. Figure 2.3 shows that women were more regretting of sterilization and reported bad quality of care if it was performed in the public facility (16.3%) in NFHS-4. The sterilization regret due to bad quality of care and performed in public facilities had also increased from NFHS-1 to NFHS-4, also the regret had increased in the private facilities due to perceived bad quality of care.

To provide the latest scenario of sterilization regret among ever married women, we have provided an estimates of the odds of women who regretted their decision of sterilization in NFHS-IV (2015–16) by different covariates. Table 2 presented the unadjusted and adjusted logistic odds ratio of sterilization regret among ever-married women in the latest round of NFHS (2015–16). Controlling for all the factors listed in the method section, the sterilization regret among states with TFR less than 2.1 was found to be high (AOR-1.26, 95% CI (1.19–1.36)) than those who have TFR more than equal to 2.1. Also, sex composition of the living children was found to be a significant factor, where women were found to be regretting more if they have only daughters (AOR-1.24, 95% CI (1.15 1.34)). It was found that the compensation received for sterilization (new question added in NFHS-4) was found be a very important factor to determine the sterilization regret. The sterilization regret was less likely among women who have received their compensation for sterilization (AOR-0.90; 95% CI (0.86–0.94)) than those who have not received compensation. Also, in a private health facility, the regret was found to be less (AOR-0.86; 95% CI- (0.81–0.92)) than sterilization performed at the public health facility. Quality of care displayed a significant importance in the regret; women were more likely to report sterilization regret when the quality of care was bad compared to very good quality of care during and post-sterilization (AOR-2.31; 95% CI- (1.89–2.82)).

**Table 2:** Unadjusted and adjusted odds ratios (with 95% CI) from binary logistic regressions examining the sterilization regret among ever married women by selected covariates in India, NFHS-2015-16

	Column 1 (OR (95% CI))	Column 2 (AOR (95% CI))
**Region**		
TFR more than 2.1®	1.00	1.00
TFR less than equal to 2.1	1.12***(1.08 1.16)	0.99 (0.95 1.03)
**Place of residence**		
Urban®	1.00	1.00
Rural	0.89***(0.86 0.93)	0.92***(0.88 0.97)
**Caste**		
Schedule caste®	1.00	1.00
Schedule tribe	1.13***(1.06 1.20)	1.07*(1 1.14)
OBC	1.07***(1.02 1.13)	1.02 (0.96 1.07)
Others	1.02 (0.96 1.08)	0.97 (0.91 1.04)
**Religion**		
Hindu®	1.00	1.00
Muslim	1.50***(1.40 1.60)	1.38***(1.28 1.49)
Christin	1.58***(1.45 1.73)	1.53***(1.39 1.68)
Others	0.76***(0.68 0.84)	0.78***(0.70 0.87)
**Wealth quintile**		
Poorest®	1.00	1.00
Poorer	1.05*(0.99 1.12)	1.02 (0.96 1.09)
Middle	1.06*(1.00 1.12)	0.96 (0.90 1.03)
Richer	1.12***(1.05 1.19)	0.97 (0.90 1.04)
Richest	1.03 (0.97 1.10)	0.88***(0.81 0.95)
**Age of women**		
15–19®	1.00	1.00
20–24	0.57 (0.31 1.06)	0.71 (0.36 1.39)
25–29	0.57 (0.31 1.04)	0.69 (0.36 1.35)
30–34	0.54**(0.29 0.99)	0.66 (0.34 1.29)
35–39	0.57 (0.31 1.04)	0.71 (0.37 1.39)
40–44	0.53**(0.29 0.98)	0.69 (0.36 1.36)
45–49	0.52**(0.28 0.94)	0.68 (0.35 1.33)
**Educational status**		
No education®	1.00	1.00
Primary	1.00 (0.94 1.05)	0.98 (0.92 1.04)
Secondary	1.12***(1.07 1.16)	1.06**(1 1.11)
Higher	1.10*(1.00 1.21)	1.01 (0.91 1.13)
**Currently married**		
Yes®	1.00	–
No	0.95 (0.87 1.03)	–
**Sex composition**		
Only Son®	1.00	1.00
Only Daughter	1.42***(1.32 1.53)	1.34***(1.24 1.44)
Both	0.77***(0.74 0.80)	0.81***(0.77 0.85)
**Child loss**		
No loss®	1.00	1.00
Before Sterilization	1.09***(1.04 1.15)	1.27**(1.04 1.56)
After Sterilization	1.54**(1.01 2.33)	1.24 (0.74 2.07)
**Age at sterilization**		
< 25®	1.00	1.00
25–29	0.93***(0.89 0.97)	0.98 (0.94 1.04)
> =30	0.93***(0.89 0.97)	0.98 (0.91 1.05)
**Year since sterilization**		
< 2®	1.00	1.00
2–3	1.10**(1.02 1.19)	1.14***(1.05 1.24)
More than 3	1.10**(1.02 1.18)	1.13***(1.03 1.24)
**Parity at sterilization**		
1®	1.00	1.00
More than 2	1.97***(1.79 2.17)	1.17***(1.11 1.24)
**Told sterilization would mean no more children**		
No®	1.00	1.00
Yes	1.39***(1.32 1.46)	1.41***(1.34 1.48)
**Compensation received**		
No®	1.00	1.00
Yes	0.89***(0.85 0.92)	0.92***(0.87 0.96)
**Quality of care**		
Very good®	1.00	1.00
All right	0.72***(0.7 0.75)	0.74***(0.71 0.77)
Not so good	1.31***(1.2 1.43)	1.33***(1.22 1.46)
Bad	2.44***(2.03 2.94)	2.39***(1.96 2.91)
**Type of health facility**		
Public®	1.00	1.00
Private	1.06**(1 1.12)	0.90′***(0.84 0.96)
**Child loss overall**		
No loss®	1.00	1.00
1 child loss	1.11***(1.05 1.18)	0.97 (0.79 1.19)
2 child loss	1.02 (0.9 1.14)	0.93 (0.74 1.18)
More than 2 loss	1.10 (0.91 1.34)	Omitted

Column1 represents the univariate (unadjusted) logistic odds ratio with 95% Confidence Interval

Column2 represents the multivariate (adjusted) logistic odds ratio with 95% Confidence Interval

***p < 0.01,**p < 0.05, *p < 0.1,

## Discussions

In the recent past, public health activists had focused their interest in the quality of care provided in the family planning programmes in the developing world. In a country like India where most of the population belonged to the rural areas, the quality of care had become increasingly prominent [3]. In 1988, Bruce had given a framework of quality of care, which defines quality as index of six elements. First is the choice of methods which includes, number of the methods offered to the receivers on consistent basis and their intrinsic variability. Second, Information given to clients consists of complete information should be provided to clients, side effects of method adopted, how to use it efficiently, what can they expect from the providers (advice, support, supply and referral to other services). Koeing et al. [22], in his systematic review on quality of care in the family planning programme, concluded that in India, the women were not counselled adequately about the other methods of contraceptives nor they were educated about the possible warning signs and side effects after the operation. A study conducted in Bihar and West Bengal also cited that the midwives were rarely discussed about the side-effects related to a contraceptive method [53]. A study conducted by Jain [19], concluded that women receive minimum information about the sterilization method, which may have contributed to the sterilization regret among them. Third, Provider competence also one of the important pillar of quality of care which referred to the skills of the providers. Fourth, Intra-personal relationship among the clients and providers. Previous studies conducted in India, provided a significant evidence, women experienced harsh and derogatory treatment while seeking family planning services in the public sector [9, 11, 36]. Fifth, Re-contact and follow up mechanism which referred to the continuity with the clients who had received services to promote program’s interest. In our study we did not able to capture whether women were followed or re-contact post their sterilization, as no question were asked related to it, but literature quotes that follow-up of clients significantly contributes to the un-happiness of the client [43, 49]. Finally, appropriate constellation of services the sixth element means the range of family planning services available to clients according to their needs. Though, the fertility programme had shaped from the recent past by accessing the range of demand and supply factors, but still the quality issues still to be need attention. Many recent studies, exploring the quality issues in family planning programme was well studied and documented that still India lacks behind in providing a safe, accessible and affordable services in the routine family planning operations [12, 22, 27, 32, 45, 46, 48]. Around 1434 deaths occur due to sterilization in the country during the years 2003 to 2012, with the maximum number in 2009, which crosses the mark of 247 deaths [50]. Due to the high rates of deaths in the sterilization camps, in 2005, the Supreme Court issued guidelines for mass vasectomy in the country; different states were asked to form a panel of qualified doctors to conduct operations in the camps and also directed doctors to counsel women and ensure the women if something went wrong during the operation. They were also asked to take the informed consent from the women about the operation (Supreme Court of India. Laws (SC)-2005–3-159, [51]). The Supreme Court of India also passed a rule that ensured the standard quality of care during these operations and compensation for families who died due to the botched operations [41], but still, a substantial number of reports publishing and addressing the same issue. The situation had become worse in a decade, which led to the ban on the sterilization camp in the country by Supreme court in 2016 [25, 47] and the supreme court asked different states that within 3 years the sterilization camps should be discontinued.

Still, a long path had to be made by India to improve the quality of standard in their public health facilities to provide good quality health care to the individuals. A study conducted in Bihar indicates an inferior quality of services provided to the women which correlates with the disappointment among them because of the sterilization operation [1]. A report by ICRW in Bihar, India accessed the quality maintained in the public facilities, and reported bad quality of care in the public hospitals of Bihar, where they reported that the hospitals were overcrowded and also the patients were not informed about the side effects associated with the procedure of female sterilization [1]. It was found that the women were neither checked before getting discharged, nor they were given necessary information on rest, bath, and follow-up visits. The females were neither informed about the side effects associated with the process nor were told about the other methods of family planning methods [2]. One high-profile event that took place in Chhattisgarh, 2014 had revealed the darkest situation of India of quality of care in sterilization operation in “**public hospitals**”, in which almost 83 women were gone for sterilization and the procedure was done in less than 6 h, which led to the death of 13 women in the sterilization camp in Bilaspur [24].

The general picture that emerged from the analysis can be summed as over time the regret associated with the sterilization had increased to approximately 3 % points from NFHS-3 to 4. Various covariates had significantly contributed to the sterilization regret, but among all, quality of care and sterilization did in the type of facility had contributed most to the sterilization regret.

The predicted probability confirms that women who experience a lousy quality of care at the time of sterilization and operated in the public facility were found to more regretting on their decision of sterilization. Also, the regret due to quality of care in private facilities had also increased over the time. The health facilities had seriously strayed from improving the health and well-being of women in providing family planning methods.

Also, the study hinted that the compensation for sterilization also correlated to the regret. Many types of research provided significant evidence that most of the sterilization were conducted to get the compensation out of the operation, the compensation amount differ in all the states based on the fertility rate in the state. In the high focus state, the compensation amount was about 15.5 USD per vasectomy and 8.5 USD per tubectomy, whereas, in the non-high focus state, the compensation received from tubectomy was 3.5 USD (for non-Below Poverty line, Scheduled Caste and tribe) all vasectomy compensation was the same as in the high focus state [33]. A case study in Rajasthan mentioned that because of the massive incentives, husbands were pushing their wives for the routine process [35]. In spite of the ban by Supreme court on sterilization camps, camps do hold in Rajasthan, and massive compensations were being offered to both men and women [7], and this lucrative inducement made women undergo sterilization which eventually resulted in a situation of grief.

The study had also pertained so some limitations that though the study tried to see the contribution of perceived quality of care in the sterilization operations on the sterilization, but failed to compute the quality of care in the family planning operations based on the Bruce/Jain Framework, as the data set failed to provide information related to all the six elements.

## Conclusions

The study concluded that around 7 % of women were regretting about their decision of sterilization according to the latest round of NFHS. Though many socio-economic and demographic factors had influenced the regret, the poor quality of care provided in the sterilization contributed maximum to the regret from 1992 to 2015. This calls for the need to standardize the facilities provided in every health facility so to minimize the dissatisfaction among the users for the routine process. Also, the government should plan out the policies related to following up of the women post the family planning operations to avoid any complication, which can help to minimize the complication or death attributed to sterilization.

The data also hinted the regret after a loss of a child (though not significant), so government should make efforts to motivate people for adopting more temporary methods of family planning especially among those who have no children or one child. Accredited Social Health Activist (ASHA) workers and Anganwadi workers are the first to come in contact with the women during the trimester, they can be motivated to encourage women to adopt more of temporary methods as they are reversible, as previous literature suggests that the least information is provided by the health service provider on the sterilization [19]. So, if the health care provider can provide all the information and provide method choice to the women, they can able to decide the best suitable method for them, so which can significantly minimize the regret percentage in the country. Social media advertisements can also become a great medium to help the couple to choose what method they should adopt for limiting or spacing their family size. There should also be a focus on male sterilization which is gradually disappearing from society as it is less complicated and can be recovered quickly compared to female sterilization.

## Supplementary information

**Additional file 1: Table 1**. Percentage Distribution of Sterilized women and percent regret among different states of India, NFHS-III. **Table 2**. Percentage Distribution of Sterilized women and percent regret among different states of India, NFHS-IV (2015–2016). **Table 3**. Proportion of Mean of sterilization regret by different background Variables, NFHS-III (2005–2006) and NFHS-IV (2015–2016).

## Data Availability

The data is available online on the website and can be downloaded. International Institute for Population Sciences, Mumbai was the nodal agency for NFHS-4; therefore, being the faculty and students of this institute, we have accessed the data from institute’s datacenter.

## Abbreviations

UN: United Nations
SC: Schedule Caste
ST: Schedule Tribe
OBC: Other backward Classes
NFHS: National Family Health Survey

## Footnotes

1 NFHS-2 (1998–99) was not used for analysis as the survey did not collect data on sterilization regret

2 The classification of geographic region is based on the Total fertility rate (TFR) as reported in NFHS-1 Report 1992–93; NFHS-3 Report 2005–06; NFHS-4 Report 2015–16.

## References

[1] Achyut P, Nanda P, Khan N, Verma R (2014). Quality of care in provision of female sterilization in Bihar: A Summary Report.

[2] Andrew M. Inside India’s female sterilization camps. Bloomberg Businessweek. 2013 Retrieved from https://www.bloomberg.com/news/articles/2013-06-20/inside-indias-female-sterilization-camps.

[3] Bansal A, Dwivedi LK (2018). Utilization of maternal and child health services: an initiation to contraception use (Unpublished M.Phil. Dissertation).

[4] Biswas S. India’s dark history of sterilization. BBC News World Edition. 2014 Retrieved from https://www.bbc.com/news/world-asia-india-30040790.

[5] Bruce J (1990). Fundamental elements of the quality of care: a simple framework. Stud Fam Plan.

[6] Chi IC, Jones DB (1994). Incidence, risk factors, and prevention of poststerilization regret in women: an updated international review from an epidemiological perspective. Obstet Gynecol Surv.

[7] Despite SC Ban, sterilization camps to be held in Rajasthan? Times of India. 2017 Retrieved from https://timesofindia.indiatimes.com/city/jaipur/despite-sc-ban-sterilization-camps-to-be-held-in-state/articleshowprint/59507806.cms.

[8] Donabedian A (1988). The quality of care: how can it be assessed?. JAMA..

[9] Ganatra BR, Coyaji KJ, Rao VN (1998). Too far, too little, too late: a community-based case-control study of maternal mortality in rural west Maharashtra, India. Bull World Health Organ.

[10] Gray A (1996). Regret after sterilization: can it be averted? Policy Dialogue, Dhaka, Population Council.

[11] Gupta JA. 'People like you never agree to get it': an Indian family planning clinic. Reprod Health Matters. 1993;1(1):39–43.

[12] Gwatkin DR. Political will and family planning: the implications of India’s emergency experience. Popul Dev Rev. 1979:29–59.

[13] Hapugalle D, Janowitz B, Weir S, Covington DL, Wilkens L, Aluvihare C. Sterilization regret in Sri Lanka: a retrospective study. Int Fam Plan Perspect. 1989:22–8.

[14] Henshaw SK, Singh S (1986). Sterilization regret among U.S. couples. Fam Plan Perspect.

[15] Hillis SD, Marchbanks PA, Tylor LR, Peterson HB (1999). Poststerilization regret: findings from the United States collaborative review of sterilization. Obstet Gynecol.

[16] International Institute for Population Sciences (IIPS) and ICF (2017). National family health survey (NFHS-4), 2015–16.India.

[17] International Institute for Population Sciences (IIPS) and Macro International (2007). National Family Health Survey (NFHS-3), 2005–06, volume 1.

[18] Jain AK. Fertility reduction and the quality of family planning services. Stud Fam Plan. 1989:1–16. 2652381

[19] Jain AK (2016). Examining progress and equity in information received by women using a modern method in 25 developing countries. Int Perspect Sex Reprod Health.

[20] Kandala N, Fahrmeir L, Klasen S, Priebe J (2009). Geo-additive models of childhood undernutrition in three sub-Saharan African countries. Popul Space Place.

[21] Kim SH, Shin CJ, Kim JG, Moon SY, Lee JY, Chang YS (1997). Microsurgical reversal of tubal sterilization: a report on 1,118 cases. Fertil Steril.

[22] Koenig MA (2003). The impact of quality of care on contraceptive use: evidence from longitudinal data from rural Bangladesh.

[23] Koenig MA, Foo GH, Joshi K (2000). Quality of care within the Indian family welfare programme: a review of recent evidence. Stud Fam Plan.

[24] Krishnan U, Pradhan B. Doctor Used Infected Tools on Indian Women as 10 Dead. Bloomberg Businessweek. 2014. Retrieved from https://www.bloomberg.com/news/articles/2014-11-11/eight-women-dead-after-india-mass-sterilization-goes-awry.

[25] Kundan P. Supreme court orders ban on mass sterilization. Down Earth. 2016.

[26] Ledbetter R (1984). Thirty years of family planning in India. Asian Surv.

[27] Loha E, Asefa M, Jira C, Tesema F (2004). Assessment of quality of care in family planning services in Jimma Zone, Southwest Ethiopia. Ethiop J Health Dev.

[28] Machado KMDM, Ludermir AB, Costa AMD (2005). Changes in family structure and regret following tubal sterilization. Cad Saúde Pública.

[29] Matthews Z, Padmadas SS, Hutter I, McEachran J, Brown JJ (2009). Does early childbearing and a sterilization-focused family planning programme in India fuel population growth?. Demogr Res.

[30] McGonigle KF, Huggins GR (1990). Tubal sterilization: epidemiology of regret. Contemp Obstetand Gynecol.

[31] Mishra V, Roy TK, Retherford RD (2004). Sex differentials in childhood feeding, health care, and nutritional status in India. Popul Dev Rev.

[32] Mohammad-Alizadeh S, Wahlström R, Vahidi R, Johansson A (2009). Women's perceptions of quality of family planning services in Tabriz, Iran. Reprod Health Matters.

[33] MOHFW (n.d.). Annual Report 2013-2014. new Delhi https://www.mohfw.gov.in/sites/default/files/Chapter1915.pdf.

[34] MOSPI. Women and Men in India (A statistical compilation of Gender related Indicators in India). New Delhi; 2018. Retrieved from http://www.mospi.gov.in/sites/default/files/publication_reports/WomenandMeninIndia2018.pdf.

[35] Murali K (2011). Rajasthan introduces sterilization incentive scheme.

[36] Nataraj S (1994). The magnitude of neglect: women and sexually transmitted diseases in India. Private Decisions, Public Debate: Women, Reproduction and Population.

[37] Nivasan K (1998). Population policies and programmes since independence: a saga of great expectations and poor performance. State Of Natural And Human Resources (2 Vols. Set).

[38] Percher J (2016). Too much of a good thing? Female sterilization in India: a literature review.

[39] Petta CA, Bahamondes L, Hidalgo M, Faundes A, Bedone AJ, Faundes D (1995). Follow-up of women seeking sterilization reversal: a Brazilian experience. Adv Contracept.

[40] Pradhan MR, Dwivedi LK (2019). Changes in contraceptive use and method mix in India: 1992–92 to 2015–16. Sex Reprod Healthc.

[41] Pulla P (2014). Why are women dying in India’s sterilization camps?. BMJ..

[42] Ram F, Roy TK (2004). Comparability issues in large sample surveys-some observations. Population, health and development in India-changing perspectives. International Institute for Population Sciences, Mumbai.

[43] Ramanathan M, Dilip TR, Padmadas SS (1995). Quality of care in laparoscopic sterilisation camps: observations from Kerala, India. Reprod Health Matters.

[44] Ramanathan M, Mishra US (2000). Correlates of female sterilization regret in the southern states of India. J Biosoc Sci.

[45] RamaRao S, Lacuesta M, Costello M, Pangolibay B, Jones H. The link between quality of care and contraceptive use. Int Fam Plan Perspect. 2003:76–83. 10.1363/ifpp.29.076.03 12783771

[46] RamaRao S, Mohanam R (2003). The quality of family planning programs: concepts, measurements, interventions, and effects. Stud Fam Plan.

[47] Sandhya S (2016). Why hundreds of women have died in the government’s horrific sterilisation camps. Scroll.In.

[48] Sanogo D, RamaRao S, Jones H, N'diaye P, M'bow B, Diop CB. Improving quality of care and use of contraceptives in Senegal. Afr J Reprod Health. 2003:57–73. 14677301

[49] Shariff A, Visaria P (1991). Family planning programme in Gujarat: a qualitative assessment of inputs and impact.

[50] Sourjya B. Death due to sterilisation nothing new in India. Hindustan Times. 2014; Retrieved from https://www.hindustantimes.com/india/death-due-to-sterilisation-nothing-new-in-india/story-sp5TE7RZSMfrV7ACFY3DIM.html.

[51] Supreme Court of India (2015). Laws (SC)-2005–3-159.

[52] United Nation (2015). The Millennium development goals report 2015.

[53] Verma RK, Roy TK (1999). Assessing the quality of family planning service providers in four Indian states. Improving Quality of Care in India’s Family Welfare Programme: The Challenge Ahead.

[54] Vieira EM, Ford NJ (1996). Regret after female sterilization among low-income women in Sao Paulo, Brazil. Int Fam Plan Perspect.

