# Supplementary material for: Sterilization regret in India: Is quality of care a matter of concern?
Source: Contracept Reprod Med. 2020 Sep 8;5:13. doi: 10.1186/s40834-020-00115-8 (PMC7487658; doi:10.1186/s40834-020-00115-8)
Supplement: Supplementary file 1 — Additional file 1: Table 1. Percentage Distribution of Sterilized women and percent regret among different states of India, NFHS-III. Table 2. Percentage Distribution of Sterilized women and percent regret among different states of India, NFHS-IV (2015–2016). Table 3. Proportion of Mean of sterilization regret by different background Variables, NFHS-III (2005–2006) and NFHS-IV (2015–2016). [file 40834_2020_115_MOESM1_ESM.docx]

**Appendix**

## **Table 1 : Percentage Distribution of Sterilized women and percent regret among different states of India, NFHS-III**

| **States** | **Percent Sterilization** | **Total No. Of Women** | **Percent Regret** | **Total No. Of Sterilized Women** |
| --- | --- | --- | --- | --- |
| **Total Fertility Rate Less than 2.1** | | | | |
| Andhra Pradesh | 52.13 | 7,128 | 4.55 | 3,325 |
| Goa | 17.35 | 3,729 | 6.78 | 1,260 |
| Himachal Pradesh | 36.69 | 2,790 | 1.89 | 870 |
| Karnataka | 44.69 | 6,008 | 7.35 | 2,703 |
| Kerala | 37.6 | 3,566 | 6.8 | 1,340 |
| Maharashtra | 39.81 | 9,034 | 2.89 | 3,344 |
| Tamil Nadu | 42.04 | 5,919 | 3.28 | 2,596 |
| Punjab | 23.14 | 3,681 | 2.44 | 858 |
| **Total Fertility Rate More than 2.1** | | | | |
| Arunachal Pradesh | 15.98 | 1,647 | 9.95 | 262 |
| Assam | 9.72 | 3,840 | 5.11 | 375 |
| Bihar | 19.67 | 3,818 | 4.51 | 788 |
| Chhattisgarh | 31.04 | 3,810 | 3.12 | 1,183 |
| Delhi | 16.97 | 3,349 | 5.3 | 593 |
| Gujarat | 33.74 | 3,464 | 4.92 | 604 |
| Haryana | 30.75 | 3,193 | 2.7 | 1,084 |
| Jharkhand | 19.06 | 2,983 | 7.52 | 597 |
| Jammu And Kashmir | 16.9 | 3,281 | 7.36 | 561 |
| Meghalaya | 5.66 | 6,427 | 20.8 | 2,263 |
| Manipur | 5.04 | 4,512 | 11.05 | 228 |
| Madhya Pradesh | 36.13 | 2,124 | 3.69 | 140 |
| Mizoram | 27.17 | 1,791 | 19.44 | 486 |
| Nagaland | 6.25 | 3,896 | 9.35 | 277 |
| Orissa | 25.29 | 4,540 | 5.56 | 1,125 |
| Rajasthan | 27.63 | 3,892 | 2.97 | 1,080 |
| Sikkim | 14.8 | 2,127 | 8.43 | 321 |
| Tripura | 13.39 | 1,906 | 3.13 | 254 |
| Uttaranchal | 23.71 | 12,183 | 5.78 | 1,735 |
| Uttar Pradesh | 13.34 | 2,953 | 4.13 | 691 |
| West Bengal | 26.77 | 6,794 | 4.04 | 1,630 |
| Total | **29.55** | **124,385** | **4.38** | **32,573** |

## **Table 2 : Percentage Distribution of Sterilized women and percent regret among different states of India, NFHS-IV (2015-2016)**

| **States** | **Percent Sterilization** | **Total No. Of Women** | **Percent Regret** | **Total No. Of Sterilized Women** |
| --- | --- | --- | --- | --- |
| **Total Fertility Rate Less than 2.1** | | | | |
| Andhra Pradesh | 62.78 | 17,995 | 6.31 | 9,218 |
| Arunachal Pradesh | 10.67 | 14,294 | 16.57 | 1,033 |
| Goa | 16.23 | 1,696 | 1.25 | 174 |
| Himachal Pradesh | 34.52 | 9,929 | 2.63 | 2,575 |
| Karnataka | 48.81 | 26,291 | 8.47 | 10,315 |
| Kerala | 45.38 | 11,033 | 8.75 | 3,844 |
| Maharashtra | 50.32 | 29,460 | 3.71 | 12,174 |
| Tamil Nadu | 49.22 | 28,820 | 11.59 | 10,653 |
| Punjab | 37.12 | 19,484 | 3.63 | 5,452 |
| Delhi | 19.63 | 5,914 | 5.32 | 759 |
| Haryana | 37.83 | 21,654 | 5.9 | 6,688 |
| Jammu And Kashmir | 23.96 | 23,800 | 14.3 | 3,406 |
| Gujarat | 33.24 | 22,932 | 7.47 | 6,213 |
| Odisha | 27.68 | 33,721 | 6.67 | 7,309 |
| Sikkim | 16.95 | 5,293 | 6.68 | 609 |
| Tripura | 13.7 | 4,804 | 1.94 | 540 |
| Uttarakhand | 27.34 | 17,300 | 5.09 | 3,951 |
| West Bengal | 28.94 | 17,668 | 8.33 | 4,328 |
| **Total Fertility Rate More than 2.1** | | | | |
| Assam | 9.21 | 28,447 | 4.86 | 1,946 |
| Bihar | 20.39 | 45,812 | 5.32 | 8,245 |
| Chhattisgarh | 44.22 | 25,172 | 7.18 | 8,014 |
| Jharkhand | 30.18 | 29,046 | 8.41 | 6,725 |
| Madhya Pradesh | 41.47 | 62,803 | 6.71 | 20,191 |
| Manipur | 2.95 | 13,593 | 16.32 | 279 |
| Meghalaya | 5.78 | 9,202 | 4.95 | 383 |
| Mizoram | 15.57 | 12,279 | 14.6 | 1,315 |
| Nagaland | 8.23 | 10,790 | 10.44 | 683 |
| Rajasthan | 40.37 | 41,965 | 5.46 | 13,224 |
| Uttar Pradesh | 16.83 | 97,661 | 6.87 | 12,268 |
| **Total** | **35.74** | **699,686** | **6.92** | **165,568** |

**Table 3: Proportion of Mean of sterilization regret by different background Variables, NFHS-III (2005-2006) and NFHS-IV (2015-2016)**

|  | **NFHS-III (2005-2006)** | | **NFHS-IV (2015-2016)** | |  |
| --- | --- | --- | --- | --- | --- |
| **Regret** | **β_1_** | **S.E(1)** | **β _2_** | **S.E(2)** | **Z=( β _2_- β_1_)/(√S.E(1)+(S.E(2)** |
| **State** |  |  |  |  |  |
| With TFR Less Than 2.1® | 0.00 |  | 0.00 |  |  |
| With TFR More Than 2.1 | 0.278 | 0.056 | -0.033 | 0.022 | -1.116 |
| **Caste** |  |  |  |  |  |
| Schedule Caste/Tribe® | 0.000 |  | 0.000 |  |  |
| Others | -0.227 | 0.062 | -0.033 | 0.022 | 0.668 |
| **Religion** |  |  |  |  |  |
| Hindu® | 0.000 |  | 0.000 |  |  |
| Muslim | 0.457 | 0.097 | 0.319 | 0.039 | -0.374 |
| Others | 0.534 | 0.081 | 0.123 | 0.037 | -1.200 |
| **Place Of Residence** |  |  |  |  |  |
| Urban® | 0.000 |  | 0.000 |  |  |
| Rural | 0.057 | 0.063 | -0.098 | 0.025 | -0.526 |
| **Educational Status** |  |  |  |  |  |
| No Education® | 0.000 |  | 0.000 |  |  |
| Primary | 0.080 | 0.076 | -0.007 | 0.029 | -0.269 |
| Secondary | 0.202 | 0.072 | 0.083 | 0.025 | -0.381 |
| Higher | 0.086 | 0.154 | 0.047 | 0.055 | -0.086 |
| **Economic Status** |  |  |  |  |  |
| Poorest® | 0.000 |  | 0.000 |  |  |
| Poorer | 0.058 | 0.112 | 0.017 | 0.032 | -0.106 |
| Middle | 0.127 | 0.108 | -0.045 | 0.034 | -0.456 |
| Richer | 0.138 | 0.111 | -0.047 | 0.036 | -0.481 |
| Richest | 0.069 | 0.123 | -0.172 | 0.042 | -0.592 |
| **Total Child Lost Post Sterilization** |  |  |  |  |  |
| No Loss® | 0.000 |  | 0.000 |  |  |
| Male Loss | 0.140 | 1.046 | 0.081 | 0.304 | -0.050 |
| Female Loss | -0.328 | 1.028 | 0.052 | 0.398 | 0.318 |
| **Child Composition Of Living Children** |  |  |  |  |  |
| No Male® | 0.000 |  | 0.000 |  |  |
| 1 Male | -0.124 | 0.138 | -0.138 | 0.053 | -0.031 |
| 2+ Male | -0.467 | 0.104 | -0.384 | 0.040 | 0.219 |
| Both Male & Female Child | -0.718 | 0.091 | -0.563 | 0.036 | 0.436 |
| **Age At Sterilization** |  |  |  |  |  |
| <29® | 0.000 |  | 0.000 |  |  |
| 30-39 | -0.098 | 0.071 | -0.012 | 0.025 | 0.277 |
| 40-49 | 0.031 | 0.346 | 0.186 | 0.076 | 0.238 |
| **Year Since Sterilization** |  |  |  |  |  |
| Less Than 2® | 0.000 |  | 0.000 |  |  |
| 2-3 | 0.211 | 0.083 | 0.113 | 0.030 | -0.292 |
| 4+ | 0.234 | 0.073 | 0.036 | 0.027 | -0.623 |
| **Parity At Sterilization** |  |  |  |  |  |
| Less Than 2® | 0.000 |  | 0.000 |  |  |
| 2-3 | -0.100 | 0.197 | -0.157 | 0.059 | -0.114 |
| 4+ | -0.194 | 0.205 | -0.254 | 0.064 | -0.116 |
| **Quality Of Care Post Sterilization** |  |  |  |  |  |
| Very Good® | 0.000 | 0.000 | 0.000 | 0.000 |  |
| All Right | -0.244 | 0.058 | -0.322 | 0.021 | -0.279 |
| Poor | 0.415 | 0.118 | 0.259 | 0.045 | -0.387 |
| Bad | 1.181 | 0.237 | 0.831 | 0.101 | -0.601 |
| **Type Of Health Facility** |  |  |  |  |  |
| Public® | 0.000 |  | 0.000 |  |  |
| Private | -0.048 | 0.078 | -0.042 | 0.030 | 0.019 |
